# Active Disturbance Rejection Control via Neural Networks for a Lower-Limb Exoskeleton

**DOI:** 10.3390/s24206546

**Published:** 2024-10-11

**Authors:** Karina I. Espinosa-Espejel, Yukio Rosales-Luengas, Sergio Salazar, Ricardo Lopéz-Gutiérrez, Rogelio Lozano

**Affiliations:** 1 Department of Research and Multidisciplinary Studies, Center for Research and Advanced Studies of the National Polytechnic Institute, Mexico City 07360, Mexico; karina.espinoza@cinvestav.mx (K.I.E.-E.); yrosales@cinvestav.mx (Y.R.-L.); sesalazar@cinvestav.mx (S.S.); 2 CONAHCYT-INAOEP, San Andrés Cholula 72840, Mexico; jrlopez@inaoep.mx

**Keywords:** lower-limb exoskeleton, walking rehabilitation, Artificial Neural Networks, external disturbances

## Abstract

This article presents the design of a control algorithm based on Artificial Neural Networks (ANNs) applied to a lower-limb exoskeleton, which is aimed to carry out walking trajectories during lower-limb rehabilitation. The interaction between the patient and the exoskeleton leads to model uncertainties and external disturbances that are always present. For this reason, the proposed control considers that the non-linear part of the model is unknown and is perturbed by external disturbances, which are estimated by an active disturbance rejection control via Artificial Neural Networks. To validate the proposed approach, a numerical simulation and an experimental implementation of the ANN-Controller are developed.

## 1. Introduction

Exoskeletons are used to assist patients with motor disabilities, such as paraplegia or quadriplegia, during their therapy sessions. These robotic devices facilitate the execution of different movements to regain locomotion ability. Lower-limb exoskeletons provide benefits during walking-assisted execution. During therapy, the features of the exoskeleton are adjusted to the patient’s requirements, such as velocity and the number of walking cycles. Human–machine interaction leads to the appearance of uncertain dynamics that are not considered in the dynamical model of an exoskeleton. Moreover, there are external disturbances, such as the vibrations of a treadmill during the walking cycle, that have an impact on the exoskeleton performance. Therefore, a research area of interest is the development of controllers with active disturbance rejection [1].

The standard Active Disturbance Rejection Controller (ADRC) depends on the system model and a virtual variable that contains internal uncertainties, unmodeled dynamics, and external disturbances. The total uncertainty is estimated by an Extended State Observer (ESO), and the disturbances are rejected in real time [2]. The ADRC is a strategy that can be applied even when the precise mathematical description of the model is not available. The three parts of the scheme are the Tracking Differentiator (TD), the ESO, and the ESO-based feedback control. The TD provides the reference system, the ESO estimates the disturbances, and the ESO-based feedback control compensates the disturbances and tracks the desired trajectories. Some of the ADRC approaches developed in recent years use different types of ESO and feedback control schemes, such as linear, nonlinear, and fractional order schemes [3,4].

In [5], an active disturbance rejection controller with a linear Extended State Observer, a tracking differentiator, and a nonlinear state error feedback applied to a lower-limb exoskeleton of 2 Degrees of Freedom (DoFs) is developed, and the controller eliminates external disturbances and parameter variations. They test the controller with a sinusoidal function noise of −0.5° to +0.5° of variance with +20% parameter variation. Furthermore, they test a random disturbance between −1 to 1, a constant disturbance, and an harmonic disturbance.

The authors in [6] developed an active disturbance rejection controller based on a fast terminal sliding mode controller for a lower-limb exoskeleton, which is able to improve tracking performance and to converge to a bounded region. Moreover, a fractional proportional derivative-based active disturbance rejection control of a knee exoskeleton device for rehabilitation is developed in [7].

An active disturbance rejection controller based on the flatness property and a control Lyapunov function is presented in [8], which is implemented in an actuated ankle orthosis for paraplegic patients. The stability analysis is carried out with the Input-to-State Stability (ISS) method and an Extended State Observer (ESO), which estimates unknown disturbances, and this estimation is added to the control input. In [9], a piecewise Extended State Observer to estimate disturbance and uncertainty is implemented in a double-joint dexterous hand, which is driven by pneumatic artificial muscles. A proportional derivative controller with disturbance estimation is applied to the system to improve angle tracking and disturbance rejection precision. To verify the disturbance rejection ability, an object of 50 g is added to the first joint as an abrupt external disturbance.

Furthermore, a Robust Unknown Input Observer (RUIO) that obtains the decoupled estimation of the unknown disturbance, which is implemented in a robotic manipulator, is presented in [10]. In that paper, the authors develop a state-feedback control using the linear parameter-varying method to avoid torque saturation through gain shifting. Then, the authors in [11] use a control strategy based on an ESO and a finite time stable tracking differentiator (FTSTD) in an upper limb exoskeleton. The exoskeleton performs flexion and extension for the shoulder and elbow in the sagittal plane. Additionally, ADRC schemes are used in applications such as a fluid-driven hand rehabilitation device, which implements an ADRC based on an ESO to achieve the control purpose due to the disturbances generated by the unconscious tremors and the difficulty in obtaining the exact model [12].

Artificial Neural Networks (ANNs) are universal estimators of a given function [13]. The ANN estimates a function by updating the weights to reduce the error between the output and the desired function through a learning rule. Due to the ANN estimation ability, they are used in ADRC schemes, and some control applications implement ANN as estimators using the tracking error to update the weights. Different applications have implemented ADRC schemes based on ANN. In the field of biomedicine and rehabilitation, artificial intelligence is gaining ground. In [14], the authors propose a novel online real-time gait terrain detection algorithm from the measurements of a foot-mounted Inertial Measurement Unit (IMU), using a shallow cascaded Convolutional Neural Network and Long Short-Term Memory neural network (CNN-LSTM).

In [15], the authors employ a human-in-the-loop scheme with a combination of a Recurrent Neural Network (RNN) and an Adaptive Non-singular Fast Terminal Sliding Mode Controller (ANFTSMC) strategy to classify the user’s movements to control the trajectories of an exoskeleton. The authors in [16] present a neural PID control with application in an upper limb exoskeleton. They obtain a semi-globally stable neural PID controller and a locally asymptotically stable neural PID controller with a velocity observer and test standard weight training algorithms.

An adaptive command filtering control based on neural networks is designed in [17], which performs trajectory tracking of an arm robot with flexible pneumatic artificial muscles. A barrier term is introduced so that the tracking errors converge to neighborhoods of the origin, and these are also bounded. They obtained a continuous controller that is able to work with unmodeled dynamics, uncertainties, and multiple input constraints. The robustness of the method is validated on a humanoid arm robot, the control requires the outputs to be measurable, and noises caused by compressed air should be suppressed. In [18], the authors present an adaptive neural network based on the integral non-singular terminal sliding mode control method to perform a fixed-time position tracking control in a wheelchair upper-limb exoskeleton under external disturbance.

The authors in [19] use an ANN to optimize the tuning of PID gains in a multi-joint lower-limb exoskeleton for gait rehabilitation, and the different tuning methods are compared with each other. The ANN-based methods improve the results and reduce the steady-state errors. A lower limb of 12 DoFs is proposed in [20], which uses a controller based on the Time-Delay Estimation (TDE), a Computed Torque Control (CTC), and a Robust Adaptive Radial Basis Neural Network (RBNN) control. The TDE is used to estimate unmodeled dynamics and external disturbances. The robust adaptive compensator based on the RBNN approximates and compensates the error of the TDE. The TDE-based CTC shows improved results in comparison with the CTC and the sliding mode based on the CTC.

An adaptive neural network control based on an RBNN for motion tracking of a lower-limb exoskeleton is presented in [21], and the RBNN ensures the accuracy and robustness of the controller, and a disturbance observer eliminates the external disturbances online. In [22], the authors present a data-driven reinforcement learning control to work with different hemiplegic patients and to deal with unpredictable disturbances. A policy interaction algorithm learns the optimal control to achieve online adaptation.

Compared with other works, the ADRC based on an ANN considers a feedback controller linearization and the ANN that is capable of estimating the unknown function by using the tracking error. Moreover, the ADRC-ANN requires less information about the model and has a good learning capacity for nonlinear characteristics and external disturbances. Additionally, the ANN structure can be established by the designer; thus it properly estimates the disturbance.

The present paper proposes a robotic device for walking rehabilitation concerning the lower-limb exoskeleton (LLE). The LLE is used to assist patients with motor disability during the walking cycle, and this promotes the motor recovery. The contributions of this paper are listed as follows:

- A control scheme based on an ANN is proposed to estimate the external disturbances, the nonlinear exoskeleton model, and the human behavior. Furthermore, a feedback linearization controller is proposed to perform a smooth tracking trajectory to prevent injuries.
- The stability and robustness of the proposed approach are obtained using Lyapunov stability analysis.
- A rejection disturbance strategy ensures the feasibility and safety of the robot during walking rehabilitation, dealing with disturbances such as vibrations of the treadmill.

Unlike other works, we added a platform to move the patient from a wheelchair, and the system has elastic joints to smooth the movement and sense the intention of motion.

This paper is organized as follows: the dynamic model is presented in Section 2. Section 3 presents the design of the ANN-based controller, and the numerical simulation and experimental implementation results are reported in Section 4. Finally, the conclusions are presented in Section 5.

## 2. Exoskeleton Dynamic Model and Properties

### 2.1. System Description and Modeling

The exoskeleton used in this work was designed by the Research and Advanced Studies Center (https://umi.cinvestav.mx/ accessed on 20 September 2024). The lower-limb exoskeleton, manufactured using 6061-T6 aluminum alloy, has 6 active degrees of freedom (DoFs); 4 of them correspond to the lower limbs (2 for the knees and 2 for the hip), and two of them are part of the standing mechanism. The ankle joint is considered for future tests, and a mechanical support is attached to this joint to sustain the foot, since the first tests are performed on a healthy subject. The actuators for the hip and the knees are of rotational elastic type, which consists of harmonic drive motors with springs. A property of these actuators is the damping during the walking cycle. In order to obtain the angular position of each joint, two AMT203-V, CUI-devices, Oregon UE. absolute rotary magnetic encoders were used, one to read the angular position of the motor and one to read the angular position of the link.

**Figure 1:**
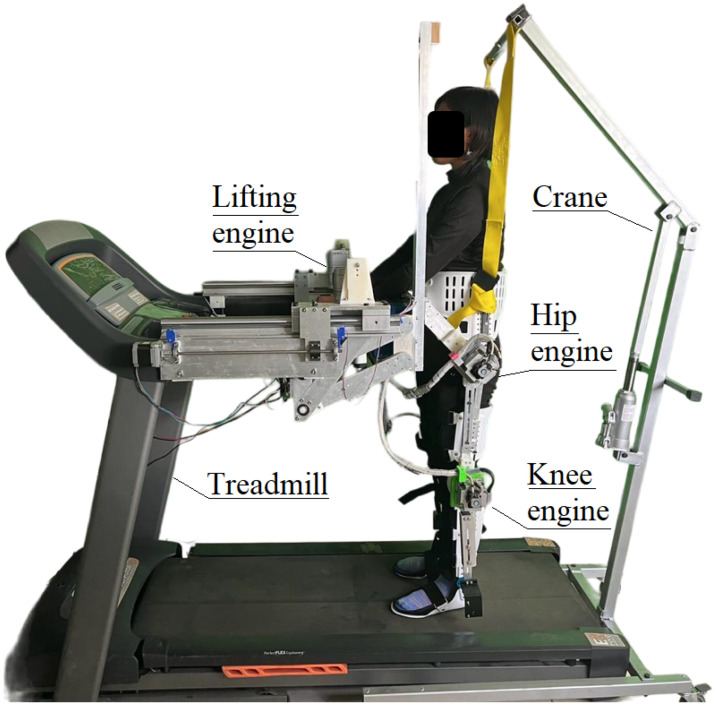
Final prototype and general distribution of actuators.

Figure 1 shows the final prototype and the general distribution of the actuators. The prototype is divided in two sections: lifting and lower-limb sections. The lifting section consists of a parallel mechanism and a safety harness attached to a crane that is used to lift up the patient. The lower-limb section consists of an exoskeleton that supports the leg, and the axis of the actuators coincides with the axis of rotation of the joints.

**Figure 2:**
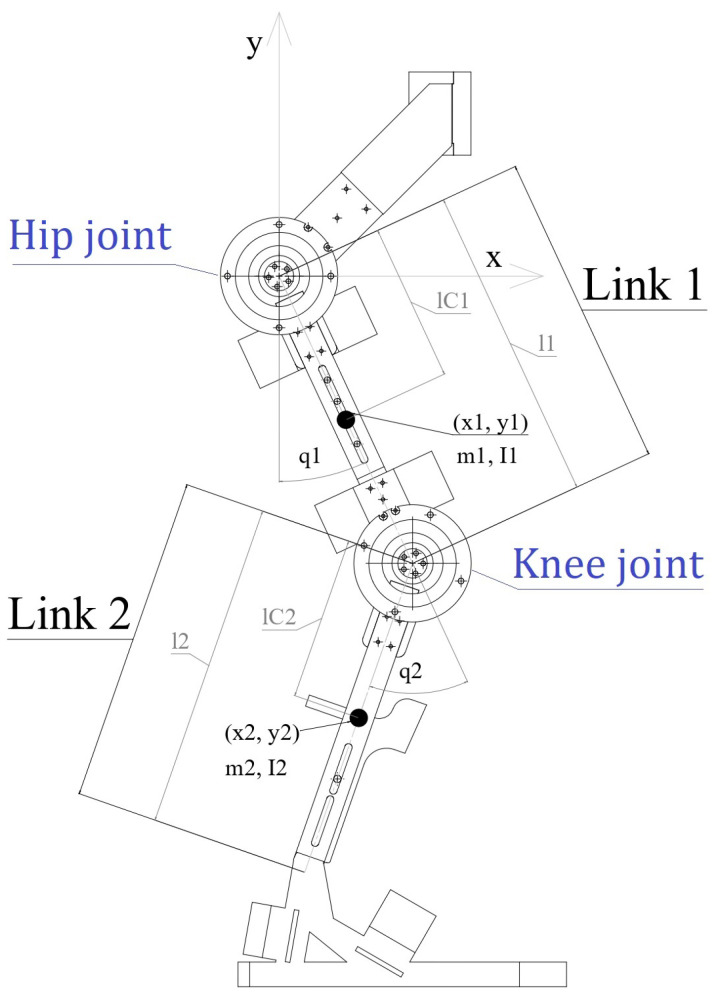
Schematic diagram and frame orientation of the lower-limb exoskeleton.

The exoskeleton has an anthropomorphic approach to simplify the adjustment to the patient’s legs, and it executes different exercises such as walking, sitting, and getting up. A schematic diagram of the exoskeleton is presented in Figure 2, in which there are two links corresponding to the thigh (link 1) and the leg (link 2) with lengths $l_{1}$ and $l_{2}$, with masses $m_{1}$ and $m_{2}$, respectively.

The angular positions $q_{1}\left(t\right)$ of the hip and $q_{2}\left(t\right)$ of the knee constitute the vector of angular positions $q\left(t\right)={\left[q_{1}\left(t\right)\;q_{2}\left(t\right)\right]}^{T}$. The dynamics of the links are actuated by the angular motion of the motors $q_{m}\left(t\right)={\left[q_{m1}\left(t\right)\;q_{m2}\left(t\right)\right]}^{T}$ through flexible joints, and the dynamics of the motors are driven by the motor torques τ. Then, the dynamic equations of the exoskeleton are as follows:

(1) $$\begin{array}{cc} \; & M\left(q\right)\ddot{q}+C\left(q,\dot{q}\right)\dot{q}+g_{m}\left(q\right)+K\left[q-q_{m}\right]=0\\ \; & J{\ddot{q}}_{m}+B{\dot{q}}_{m}-K\left[q-q_{m}\right]=\tau +d, \end{array}$$

where $M\left(q\right)\in R^{2\times 2}$ is the inertia matrix, $C\left(q,\dot{q}\right)\in R^{2\times 2}$ is the Coriolis matrix, $g_{m}\left(q\right)\in R^{2\times 1}$ is the vector of gravitational forces, and $\tau \in R^{2\times 1}$ are the torques of the actuators. *J* is a positive definite matrix with values in its diagonal equal to the product of the inertia moments of the motors and the square of the gear ratio $J=diag\{J_{1}r_{1,1}^{2},J_{2}r_{1,2}^{2}\}$. *K* is a diagonal positive definite matrix with the elastic constants on the diagonal defined as $K=diag\{k_{s1},k_{s2}\}$, *B* is a positive definite diagonal matrix, and its diagonal elements are the viscous friction parameters of each motor $B=diag\{b_{1},b_{2}\}$. Additionally, $d\in R^{2\times 1}$ is an unknown term, which contains non-modeled dynamics, external noise, and human interaction. Then, the vector d is defined as follows:

(2) $$d=\tau_{h}+D+\rho ,$$

where $\tau_{h}\;\in \;R^{2\times 1}$ is the vector of the torque generated by the human, $D\;\in \;R^{2\times 1}$ is the vector of external disturbances, and ρ is the unknown part of the model.

Furthermore, the elements of M(q), $C(q,\dot{q})$, and $g_{m}\left(q\right)$ are as follows:

(3) $$\begin{array}{cc} \; & M_{11}\left(q\right)=I_{1}+I_{2}+l_{1}^{2}m_{2}+l_{c1}^{2}m_{1}+l_{c2}^{2}m_{2}+2l_{1}l_{c2}m_{2}cos\left(q_{2}\right)\\ \; & M_{12}\left(q\right)=I_{2}+l_{c2}^{2}m_{2}+l_{c2}^{2}m_{2}+l_{1}l_{c2}m_{2}cos\left(q_{2}\right)\\ \; & M_{21}\left(q\right)=I_{2}+l_{c2}^{2}m_{2}+l_{c2}^{2}m_{2}+l_{1}l_{c2}m_{2}cos\left(q_{2}\right)\\ \; & M_{22}\left(q\right)=I_{2}+m_{2}l_{c2}^{2}\\ \; & C_{11}\left(q,\dot{q}\right)=-m_{2}l_{1}l_{c2}sin\left(q_{2}\right){\dot{q}}_{2}\\ \; & C_{12}\left(q,\dot{q}\right)=-m_{2}l_{1}l_{c2}sin\left(q_{2}\right)\left[{\dot{q}}_{1}+{\dot{q}}_{2}\right]\\ \; & C_{21}\left(q,\dot{q}\right)=m_{2}l_{1}l_{c2}sin\left(q2\right){\dot{q}}_{1}\\ \; & C_{22}\left(q,\dot{q}\right)=0\\ \; & g_{m1}\left(q\right)=\left[m_{1}l_{c1}+m_{2}l_{1}\right]gsin\left(q_{1}\right)+m_{2}gl_{c2}sin\left(q_{1}+q_{2}\right)\\ \; & g_{m2}\left(q\right)=m_{2}gl_{c2}sin\left(q_{1}+q_{2}\right) \end{array}$$

The desired trajectories corresponding to the hip and knee joints are denoted by $q_{d1}$ and $q_{d2}$, respectively.

Solving the second derivative of links and motor position, and rewriting the dynamic equations in (1), the following expressions for $\ddot{q}$ and ${\ddot{q}}_{m}$ are obtained:

(4a) $$\begin{array}{cc} \ddot{q} & =M^{-1}\left[-C\dot{q}-g_{m}-K\left(q-q_{m}\right)\right] \end{array}$$

(4b) $$\begin{array}{cc} {\ddot{q}}_{m} & =J^{-1}\left[\tau +d-B{\dot{q}}_{m}+K\left(q-q_{m}\right)\right] \end{array}$$

By using (4a) and (4b), the following expression is obtained:

(5) $$\begin{array}{cc} q^{\left(4\right)}=\; & {\ddot{M}}^{-1}\left[-C\dot{q}-g_{m}-K\left(q-q_{m}\right)\right]\\ \; & +2{\dot{M}}^{-1}\left[-C\ddot{q}-\dot{C}\dot{q}-{\dot{g}}_{m}-K\left(\dot{q}-{\dot{q}}_{m}\right)\right]\\ \; & +M^{-1}[-2\dot{C}\ddot{q}-Cq^{\left(3\right)}-\ddot{C}\dot{q}-{\ddot{g}}_{m}\\ \; & -K(\ddot{q}-J^{-1}\left[\tau +d-B{\dot{q}}_{m}+K\left(q-q_{m}\right)\right])] \end{array}$$

Furthermore, the input control τ depends on *q*, $\dot{q}$, $\ddot{q}$, $q^{\left(3\right)}$, and $q^{\left(4\right)}$. The state variables are assigned as $x_{1}=q$, $x_{2}=\dot{q}$, $x_{3}=\ddot{q}$, and $x_{4}=q^{\left(3\right)}$. Hence, the variable state vector is $x={\left[q\;\dot{q}\;\ddot{q}\;q^{\left(3\right)}\right]}^{T}\in R^{8\times 1}$, and the state space representation is as follows:

(6) $$\begin{array}{c} \dot{x}=\left[\begin{array}{c} {\dot{x}}_{1}\\ {\dot{x}}_{2}\\ {\dot{x}}_{3}\\ {\dot{x}}_{4} \end{array}\right]=\left[\begin{array}{c} \dot{q}\\ \ddot{q}\\ q^{\left(3\right)}\\ q^{\left(4\right)} \end{array}\right]=\left[\begin{array}{c} x_{2}\\ x_{3}\\ x_{4}\\ f(x)+g(x)\tau \end{array}\right], \end{array}$$

where

(7) $$\begin{array}{cc} f(x)=\; & {\ddot{M}}^{-1}\left[-Cx_{2}-g_{m}-K\left(x_{1}-q_{m}\right)\right]\\ \; & +2{\dot{M}}^{-1}\left[-Cx_{3}-\dot{C}x_{2}-{\dot{g}}_{m}-K\left(x_{2}-{\dot{q}}_{m}\right)\right]\\ \; & +M^{-1}[-2\dot{C}x_{3}-Cx_{4}-\ddot{C}x_{2}-{\ddot{g}}_{m}\\ \; & -K(x_{3}J^{-1}\left[d-B{\dot{q}}_{m}+K\left(x_{1}-q_{m}\right)\right])]\\ g(x)=\; & M^{-1}KJ^{-1} \end{array}$$

**Assumption 1.**  *The external disturbance d is bounded.*

### 2.2. Artificial Neural Network Structure for the LLE

An Artificial Neural Network (ANN) is applied to approximate the unknown function of the robot. The ANN is a universal estimator, and the structure of the neural network is as follows:

(8) $$\Delta Q=W^{T}\phi +\epsilon \;\in \;R^{2\times 1},$$

where *W*∈$R^{N\times 2}$ is the weight matrix and *N* is the number of nodes in the hidden layer.

According to the approximation property, given a continuous real function y:Ω→R in a compact set $\Omega \subset R^{n}$, by choosing $\phi_{j}$, j=1,…,N, there exists an ideal weight matrix that can approximate the function *y* with a bounded approximation error ϵ>0. The matrix of the weights is $W=[w_{jk}]$, where j={1,2,…,N} and k={1,2}; ϵ is an approximation error $\epsilon ={\left[\epsilon_{1}\;\epsilon_{2}\right]}^{T}$.

Additionally, ϕ∈$R^{N}$ is the activation function vector, and its components are as follows:

(9) $$\phi ={\left[\phi_{1}\;\phi_{2}\;\ldots \;\phi_{N}\right]}^{T}$$

In this paper, the activation function is selected to be a sigmoid function, defined by the following:

(10) $$\phi_{j}\left(v_{j}\right)=\frac{a_{j}}{1+e^{-b_{j}v_{j}}}-c_{j},$$

where $a_{j}$, $b_{j}$, and $c_{j}$ are the parameters of the sigmoid functions.

The implemented ANN has three layers, the input layer, the hidden layer with the activation functions, and the output layer. The inputs of the neural network are denoted by $v_{j}$. The ANN approximates the unknown function of the uncertainties and the non-linearities of the system. As the weight matrix W is unknown, it is estimated on-line, and it is denoted by $\hat{W}$. Moreover, an adaptive law is obtained to update the weight matrix. Therefore, the output of the on-line artificial neuron is as follows:

(11) $$\begin{array}{cc} \; & \Delta \hat{Q}={\hat{W}}^{T}\phi \;\in \;R^{2\times 1} \end{array}$$

**Remark 1.**  *We consider that the next parameters are bounded; the approximation error $\|\epsilon \|<\epsilon_{N}$, the weight matrix $\left\|W\right\|=W_{M}$, and the activation function ‖ϕ(v)‖≤σ. Additionally, we consider that $\epsilon_{N}$, $W_{M}$, and σ are positive constants.*

## 3. ANN-Based Control Design

In this section, we describe the design of an active disturbance rejection control via artificial neural networks for the lower-limb exoskeleton (LLE). The control objective is to track the desired trajectory such that the closed-loop system is uniformly ultimately bounded and the output of the system *q* follows the desired trajectory $q_{d}$.

**Figure 3:**
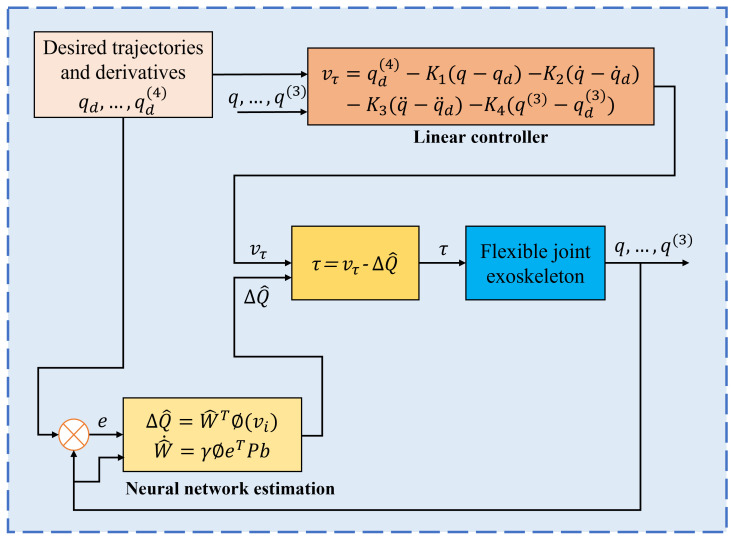
Scheme of the neural network-based control.

The diagram in Figure 3 describes the proposed control approach. The control scheme has three parts: the linear feedback control, the ANN estimation of a variable that represents the external disturbances and unmodeled dynamics (ΔQ), and the flexible joint robot model (Equation (1)). The linear feedback control includes the terms of desired position, velocity, acceleration, and the fourth derivative (jerk) of the position error.

The desired trajectories for the walking cycle are trigonometric polynomials of the 8th order [23]. Afterwards, the desired trajectory of the hip is $q_{d1}$ and the desired trajectory of the knee is $q_{d2}$, as follows:

(12) $$\begin{array}{cc} q_{d1} & =17.2sin(0.08\omega t+0.24)+69.61sin(1.00\omega t+1.72)\\ \; & +6.90sin(0.17\omega t+2)+4sin(2\omega t-1.72)+1.68sin(2.99\omega t-0.01)\\ \; & +1.63sin(0.36\omega t+2.31)+0.70sin(0.44\omega t+4.24)+48.17sin(1.01\omega t+4.81)\\ q_{d2} & =44.7sin(0.08\omega t+0.36)+21.13sin(0.99\omega t-2.92)\\ \; & +16.07sin(2\omega t-0.94)+20.08sin(0.15\omega t+2.44)+4.37sin(2.99\omega t-0.02)\\ \; & +20.92sin(0.34\omega t+2.98)+18.45sin(0.35\omega t+6)+1.27sin(3.98\omega t-0.65), \end{array}$$

where ω is the frequency of the walking cycle.

### 3.1. ANN-Based Control Design

In order to design the ANN-based control, the auxiliary linear dynamics are proposed as follows:

(13) $$\begin{array}{c} {\hat{q}}^{\left(4\right)}=\tau \end{array}$$

The error between the auxiliary dynamics and the exoskeleton dynamics is denoted as $\Delta Q=q^{\left(4\right)}-{\hat{q}}^{\left(4\right)}$ and is described as follows:

(14) $$\begin{array}{cc} \Delta Q=\; & {\ddot{M}}^{-1}\left[-C\dot{q}-g_{m}-K\left(q-q_{m}\right)\right]\\ \; & +2{\dot{M}}^{-1}\left[-C\ddot{q}-\dot{C}\dot{q}-{\dot{g}}_{m}-K\left(\dot{q}-{\dot{q}}_{m}\right)\right]\\ \; & +M^{-1}[-2\dot{C}\ddot{q}-Cq^{\left(3\right)}-\ddot{C}\dot{q}-{\ddot{g}}_{m}\\ \; & -K(\ddot{q}-J^{-1}\left[\tau +d-B{\dot{q}}_{m}+K\left(q-q_{m}\right)\right])]-\tau \end{array}$$

Then,

(15) $$\begin{array}{cc} \; & q^{\left(4\right)}={\hat{q}}^{\left(4\right)}+\Delta Q\\ \; & q^{\left(4\right)}=\tau +\Delta Q \end{array}$$

A control input is proposed as follows:

(16) $$\tau^{*}=v_{\tau }-\Delta Q$$

Substituting the above into (15) leads to the following:

(17) $$\begin{array}{cc} \; & q^{\left(4\right)}=v_{\tau } \end{array}$$

Considering the state variable assignment proposed in Section 2.1: $x_{1}=q$, $x_{2}=\dot{q}$, $x_{3}=\ddot{q}$ and $x_{4}=q^{\left(3\right)}$, and Equation (17), it follows:

(18) $$\begin{array}{cc} \; & {\dot{x}}_{1}=x_{2}\\ \; & {\dot{x}}_{2}=x_{3}\\ \; & {\dot{x}}_{3}=x_{4}\\ \; & {\dot{x}}_{4}=v_{\tau }\\ \; & y=x_{1} \end{array}$$

The input $v_{\tau }$ is proposed such that *q* follows the smooth desired trajectory $q_{d}$ when t→∞. Therefore, $v_{\tau }$ is proposed as follows:

(19) $$\begin{array}{cc} v_{\tau } & =q_{d}^{\left(4\right)}-K_{1}\left(q-q_{d}\right)-K_{2}\left(\dot{q}-{\dot{q}}_{d}\right)-K_{3}\left(\ddot{q}-{\ddot{q}}_{d}\right)-K_{4}\left(q^{\left(3\right)}-q_{d}^{\left(3\right)}\right), \end{array}$$

where $K_{i}=diag\left\{k_{i},\;k_{i}\right\}$ with i=1,2,3,4 are the gain matrices such that each gain matrix is a diagonal positive definite matrix. Substituting $v_{\tau }$ from (19) into Equation (17), we have the following:

(20) $$\begin{array}{cc} q^{\left(4\right)}-q_{d}^{\left(4\right)} & =-K_{1}\left(q-q_{d}\right)-K_{2}\left(\dot{q}-{\dot{q}}_{d}\right)-K_{3}\left(\ddot{q}-{\ddot{q}}_{d}\right)-K_{4}\left(q^{\left(3\right)}-q_{d}^{\left(3\right)}\right) \end{array}$$

The error variables are defined as $\tilde{q}=q-q_{d}$, $\dot{\tilde{q}}=\dot{q}-{\dot{q}}_{d}$, $\ddot{\tilde{q}}=\ddot{q}-{\ddot{q}}_{d}$, and  ${\tilde{q}}^{\left(3\right)}=q^{\left(3\right)}-q_{d}^{\left(3\right)}$. Let us define the error vector as follows:

(21) $$e={\left[{\tilde{q}}^{T}\;{\dot{\tilde{q}}}^{T}\;{\ddot{\tilde{q}}}^{T}\;{\tilde{q}}^{(3)T}\right]}^{T}\;\in \;R^{8\times 1},$$

and the derivative of the error vector is as follows:

(22) $$\dot{e}={\left[{\dot{\tilde{q}}}^{T}\;{\ddot{\tilde{q}}}^{T}\;{\tilde{q}}^{(3)T}\;{\tilde{q}}^{(4)T}\right]}^{T}\;\in \;R^{8\times 1}$$

Using the error vector and its derivative in (21) and (22) with (20), the dynamic equation for the tracking errors is as follows:

(23) $$\begin{array}{cc} \; & \dot{e}=Ae, \end{array}$$

where $A\in R^{8\times 8}$ is a positive definite matrix given by the following:

(24) $$\begin{array}{c} A=\left[\begin{array}{cccc} 0 & I^{2\times 2} & 0 & 0\\ 0 & 0 & I^{2\times 2} & 0\\ 0 & 0 & 0 & I^{2\times 2}\\ -K_{1} & -K_{2} & -K_{3} & -K_{4} \end{array}\right] \end{array}$$

The error between the exoskeleton model and the linear system ΔQ is assumed to be unknown. This error is estimated by a neural network, and the estimation $\Delta \hat{Q}$ has the form of Equation (11).

Therefore, using the neural network estimation, the actual control law τ is as follows:

(25) $$\tau =v_{\tau }-\Delta \hat{Q},$$

where $v_{\tau }$ is given in Equation (19) and $\Delta \hat{Q}$ is the estimation given by (11). Substituting the actual control law in Equation (15), we have the following:

(26) $$q^{\left(4\right)}=v_{\tau }+\Delta Q-\Delta \hat{Q}$$

Since the output of the neural network is $\Delta Q=W^{T}\phi \left(v\right)+\epsilon$ and the estimation is $\Delta \hat{Q}={\hat{W}}^{T}\phi \left(v\right)$, then the estimation error $\Delta \tilde{Q}$ is as follows:

(27) $$\Delta \tilde{Q}=\Delta Q-\Delta \hat{Q}=W^{T}\phi \left(v\right)+\epsilon -{\hat{W}}^{T}\phi \left(v\right)={\tilde{W}}^{T}\phi \left(v\right)+\epsilon ,$$

where the estimation error of the weights $\tilde{W}$ is as follows:

(28) $$\tilde{W}=W-\hat{W}\in R^{N\times 2}$$

Therefore, Equation (26) becomes the following:

(29) $$q^{\left(4\right)}=v_{\tau }+{\tilde{W}}^{T}\phi \left(v\right)+\epsilon$$

Substituting $v_{\tau }$ from Equation (19) in the above equation, the error dynamics are as follows:

(30) $$\begin{array}{cc} q^{\left(4\right)}-q_{d}^{\left(4\right)}= & -K_{1}\left(q-q_{d}\right)-K_{2}\left(\dot{q}-{\dot{q}}_{d}\right)-K_{3}\left(\ddot{q}-{\ddot{q}}_{d}\right)\\ \; & -K_{4}\left(q^{\left(3\right)}-q_{d}^{\left(3\right)}\right)+{\tilde{W}}^{T}\phi \left(v\right)+\epsilon \end{array}$$

Using $\Delta \tilde{Q}={\tilde{W}}^{T}\phi \left(v\right)+\epsilon$, the error vector (21), and its derivative (22), the error dynamics in matrix form are as follows:

(31) $$\dot{e}=Ae+B\Delta \tilde{Q},$$

where *A* has the form of (24) and $B={\left[0^{2\times 2}\;0^{2\times 2}\;0^{2\times 2}\;I^{2\times 2}\right]}^{T}\;\in \;R^{8\times 2}$.

### 3.2. Stability Analysis

The stability analysis of the closed-loop system is developed in the present section.

**Theorem 1.**  *Consider the closed-loop system (31), the control law (25), and the adapting law as $\dot{\hat{W}}=\gamma \phi \left(x\right)e^{T}PB$ based on [24]. Then, for any bounded initial conditions, the closed-loop system signals remain bounded and the steady-state tracking error converges to a neighborhood of the origin by choosing the parameters adequately as in Section 3.3.*

**Proof.**  To perform the stability analysis of the closed-loop system, the following Lyapunov function candidate is proposed:

(32) $$V\left(e,\tilde{W}\right)=\frac{1}{2}e^{T}Pe+\frac{1}{2\gamma }tr\left({\tilde{W}}^{T}\tilde{W}\right),$$

where γ is a positive constant, $\tilde{W}$ denotes the weight estimation error given in (28), and $P\in R^{8\times 8}$ is a positive definite symmetric matrix that satisfies the following Lyapunov equation:

(33) $$A^{T}P+PA=-Q,$$

with $Q\geq 0\in R^{8\times 8}$ and *A* given by (24). Differentiating (32), we obtain $\dot{V}\left(e,\tilde{W}\right)$ as follows:

(34) $$\dot{V}\left(e,\tilde{W}\right)=\frac{1}{2}\left(e^{T}P\dot{e}+{\dot{e}}^{T}Pe\right)+\frac{1}{\gamma }tr\left({\tilde{W}}^{T}\dot{\tilde{W}}\right)$$

Substituting the tracking error dynamics from Equation (31), we have the following:

(35) $$\begin{array}{cc} \dot{V}\left(e,\tilde{W}\right) & =\frac{1}{2}\left(e^{T}P\left(Ae+B\Delta \tilde{Q}\right)+{\left(Ae+B\Delta \tilde{Q}\right)}^{T}Pe\right)+\frac{1}{\gamma }tr\left({\tilde{W}}^{T}\dot{\tilde{W}}\right)\\ \; & =\frac{1}{2}\left(e^{T}PAe+e^{T}PB\Delta \tilde{Q}+e^{T}A^{T}Pe+\Delta \tilde{Q}B^{T}Pe\right)+\frac{1}{\gamma }tr\left({\tilde{W}}^{T}\dot{\tilde{W}}\right)\\ \; & =\frac{1}{2}\left(e^{T}\left(PA+A^{T}P\right)e+e^{T}PB\Delta \tilde{Q}+\Delta \tilde{Q}B^{T}Pe\right)+\frac{1}{\gamma }tr\left({\tilde{W}}^{T}\dot{\tilde{W}}\right)\\ \; & =-\frac{1}{2}e^{T}Qe+e^{T}PB\left({\tilde{W}}^{T}\phi \left(v\right)+\epsilon \right)+\frac{1}{\gamma }tr\left({\tilde{W}}^{T}\dot{\tilde{W}}\right)\\ \; & =-\frac{1}{2}e^{T}Qe+e^{T}PB{\tilde{W}}^{T}\phi \left(v\right)+e^{T}Pb\epsilon +\frac{1}{\gamma }tr\left({\tilde{W}}^{T}\dot{\tilde{W}}\right) \end{array}$$

Using the trace property, we have $e^{T}PB{\tilde{W}}^{T}\phi \left(v\right)=tr\left({\tilde{W}}^{T}\phi \left(v\right)e^{T}PB\right)$. Additionally, $\tilde{W}=W-\hat{W}$; then $\dot{\tilde{W}}=-\dot{\hat{W}}$, and $\dot{V}\left(e,\tilde{W}\right)$ is as follows:

(36) $$\begin{array}{cc} \dot{V}\left(e,\tilde{W}\right) & =-\frac{1}{2}e^{T}Qe-tr\left({\tilde{W}}^{T}\phi \left(v\right)e^{T}PB\right)+e^{T}PB\epsilon +\frac{1}{\gamma }tr\left({\tilde{W}}^{T}\dot{\tilde{W}}\right)\\ \; & =-\frac{1}{2}e^{T}Qe-tr\left({\tilde{W}}^{T}\phi \left(v\right)e^{T}PB+\frac{1}{\gamma }{\tilde{W}}^{T}\dot{\tilde{W}}\right)+e^{T}PB\epsilon \\ \; & =-\frac{1}{2}e^{T}Qe-tr\left({\tilde{W}}^{T}\left(\phi \left(v\right)e^{T}PB-\frac{1}{\gamma }\dot{\hat{W}}\right)\right)+e^{T}PB\epsilon \end{array}$$

Since our aim is that $\phi \left(v\right)e^{T}PB-\frac{1}{\gamma }\dot{\hat{W}}=0$, then the updating law of the weights $\dot{\hat{W}}$ is as follows:

(37) $$\dot{\hat{W}}=\gamma \phi \left(v\right)e^{T}PB$$

Substituting (37) into (36), then the following holds:

(38) $$\dot{V}\left(e,\tilde{W}\right)=-\frac{1}{2}e^{T}Qe+e^{T}PB\epsilon$$

We consider $\lambda_{min}{\left(Q\right)\|e\|}^{2}\leq e^{T}Qe\leq \lambda_{max}\left(Q\right){\left\|e\right\|}^{2}$ and the bounds ‖B‖=$\|I^{2\times 2}{\|}_{F}\;=\;$$tr{\left(I^{T}I\right)}^{1/2}=\sqrt{2}$ with the subindex *F* that represents the Frobenius norm of a matrix, $\left\|P\right\|\leq \lambda_{max}\left(P\right)$ and $\|\epsilon \|<\epsilon_{N}$; then we have the following:

(39) $$\begin{array}{cc} \dot{V}\left(e,\tilde{W}\right) & \leq -\frac{1}{2}\lambda_{min}{\left(Q\right)\|e\|}^{2}+\left\|e\right\|\lambda_{max}\left(P\right)\sqrt{2}\epsilon_{N}\\ & =-\frac{1}{2}\left\|e\right\|\left(\lambda_{min}\left(Q\right)\left\|e\right\|-2\sqrt{2}\epsilon_{N}\lambda_{max}\left(P\right)\right) \end{array}$$

If $\lambda_{min}\left(Q\right)\left\|e\right\|\;-\;2\sqrt{2}\epsilon_{N}\lambda_{max}\left(P\right)\geq 0$ holds, then $\dot{V}\leq 0$. Therefore, the ultimate bound for ‖e‖ is [24] as follows:

(40) $$\|e\|\;=\;\frac{2\sqrt{2}\epsilon_{N}\lambda_{max}\left(P\right)}{\lambda_{min}\left(Q\right)}$$

□

### 3.3. Control Gains Tuning

The auxiliary linear dynamics of Equation (13) are proposed, and an unknown function ΔQ is estimated by the neural network to compensate the nonlinear part of the model and external disturbances. Likewise, the control law consists of a linear controller $v_{\tau }$ and the compensation of the neural network, as shown in Equation (25). Therefore, the gains of the linear controller in Equation (19) should be tuned. The gains $k_{i}$ with i=1,2,3,4 are selected such that the roots of the characteristic polynomial $\pi_{c}\left(s\right)$ are in the left semi-plane of the complex plane.

(41) $$\pi_{c}\left(s\right)=s^{4}+k_{4}s^{3}+k_{3}s^{2}+k_{2}s+k_{1}$$

We assign the characteristic polynomial to the following 4th-order polynomial with the four roots in −1, as follows:

(42) $$\pi_{d}={\left(s+1\right)}^{4}=s^{4}+4s^{3}+6s^{2}+4s+1$$

Then, the gains are $k_{4}=4$, $k_{3}=6$, $k_{2}=4$$k_{1}=1$. This dynamic behavior guaranties an over-damping response.

The following pseudocode (Algorithm 1) describes the algorithm of the neural network:
**Algorithm 1** Pseudocode of neural network algorithmInitialize the weight matrix of the neural network as $\hat{W}\left(0\right)=ones\left(N,2\right)$ since $\hat{W}\in R^{N\times 2}$For j=1 to *N*, compute (Equation (10)):
$$\phi_{j}\left(v_{j}\right)=\frac{a_{j}}{1+e^{-b_{j}v_{j}}}-c_{j}$$
to construct the vector $\phi \;\in R^{N\times 1}$Compute the output of the neuron as $\Delta \hat{Q}={\hat{W}}^{T}\phi \;\in \;R^{2\times 1}$ (Equation (11))Compute the error vector $e={\left[{\tilde{q}}^{T}\;{\dot{\tilde{q}}}^{T}\;{\ddot{\tilde{q}}}^{T}\;{\tilde{q}}^{(3)T}\right]}^{T}\;\in \;R^{8\times 1}$ (Equation (21))Set the gain values for Equation (37) as γ=2, $P=eye\left(8,8\right)\;\in R^{8\times 8}$$B=ones\left(8,2\right)\;\in R^{8\times 2}$.Compute the derivative of the weight estimation matrix (Equation (37))
$$\dot{\hat{W}}=\gamma \phi \left(v\right)e^{T}PB$$Integrate the derivative of the weight estimation matrix $\dot{\hat{W}}$ to obtain the weight estimation $\hat{W}$Output $\hat{W}$ and $\Delta \hat{Q}$

## 4. Numerical and Experimental Results

In this section, we describe the numerical and experimental results obtained by using walking trajectories and implementing the proposed approach to the exoskeleton prototype.

### 4.1. Numerical Results

For the simulation tests, two gait cycle trajectories for the hip and knee joints are used. External disturbances are introduced as sinusoidal type signals, which represent different events in walking exercises such as muscle spasms and unmodeled dynamics of the exoskeleton. The vector of initial conditions for simulation is ${\left[x_{1}\left(0\right),x_{2}\left(0\right),x_{3}\left(0\right),x_{4}\left(0\right)\right]}^{T}$$={\left[0.5,0.1,0.5,0.1,0.5,0.1,0.5,0.1\right]}^{T}$, which represents the state initial conditions. The external disturbance vector is $d={\left[0.2sin\left(t\right)\;0.5sin\left(t\right)\right]}^{T}$. Moreover, the values of the neural network are $a_{j}=2$, $b_{j}=2$, and $c_{j}=0$. The initial weight of the neural network is $\hat{W}\left(0\right)=0$, and considering a number of activation functions N=5 with only one hidden layer, the weight matrix is as follows:

(43) $$W=\left[\begin{array}{cc} w_{1,1} & w_{1,2}\\ \cdots & \cdots \\ w_{5,1} & w_{5,2} \end{array}\right]$$

**Figure 4:**
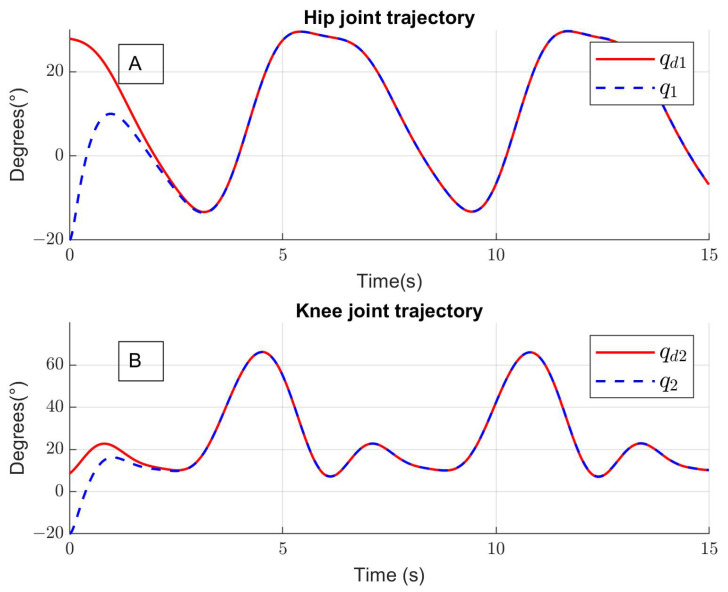
Tracking trajectories (two walking cycles) for (**A**) hip joint and (**B**) knee joint.

The parameter *N* was selected through an experimental test by reducing and increasing the number of activation functions. Then, the least number of activation functions with good performance was chosen, in this case, N=5. Figure 4 presents the trajectory tracking for two walking cycles, and the robot trajectory follows the desired trajectory with over-damping response. In Figure 4A, the red line is the desired trajectory of the hip $q_{d1}$ and the blue line is the trajectory of the hip robot joint $q_{1}$. In Figure 4B, the red line is the desired trajectory of the knee $q_{d2}$ and the blue line is the trajectory of the knee robot joint $q_{2}$ generated by the implemented control law.

**Figure 5:**
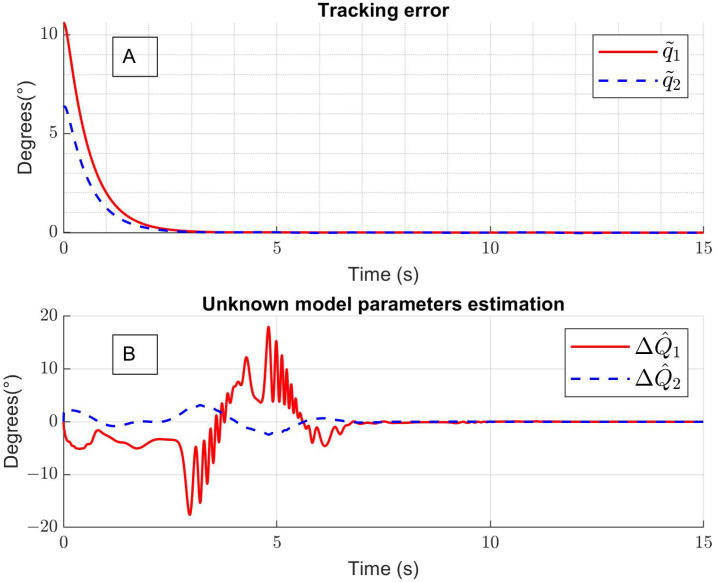
(**A**) Tracking error and (**B**) unknown parameter estimation.

**Figure 6:**
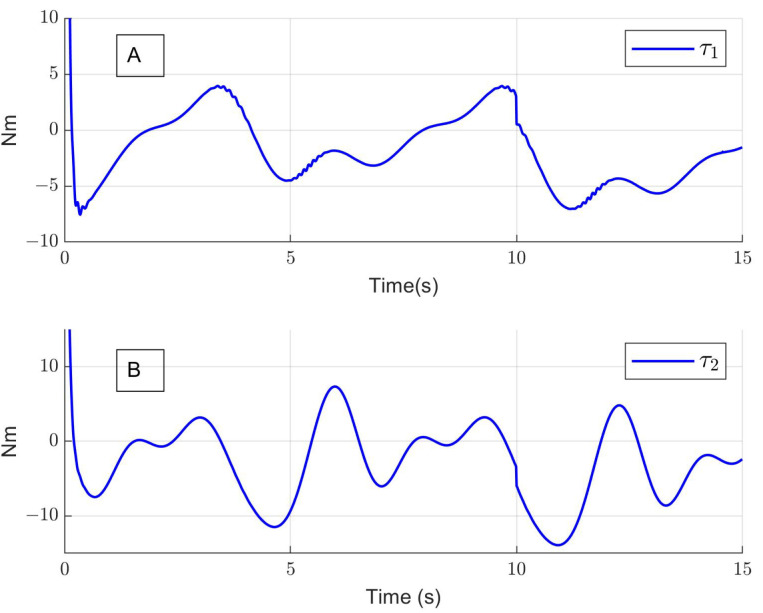
(**A**) Control input for hip joint $\tau_{1}$; (**B**) control input for knee joint $\tau_{2}$.

Figure 5A shows that the tracking error converges close to zero in approximately 2.5 s for both cases; the red line represents the hip joint error, and the blue line represents the knee joint error. Moreover, the means of absolute errors are ${\tilde{q}}_{1}=0.93$° and ${\tilde{q}}_{2}=1.33$° for the hip and the knee, respectively. Additionally, Figure 5B shows the unknown model parameter estimation by a neural network. Finally, Figure 6A and Figure 6B show the hip torque $\tau_{1}$ and the knee torque $\tau_{2}$, respectively, generated by the control implemented in the exoskeleton.

### 4.2. Experimental Setup

In this subsection, we describe the experimental setup. The control algorithm was programmed on a myRIO card from National Instruments, using LabView software 2018.

The walking exoskeleton was tested on a healthy subject of 28 years with a weight of 57 kg and a height of 1.6 m with the security conditions and an informed consent letter.

The trajectories for the hip and the knee joint were generated using the approximation of 8th-order trigonometric polynomials as described in Section 3. The walking trajectories are from a healthy patient and are generated in real time by the card as the reference trajectories for the tracking controller.

The system is operated using a graphic interface elaborated in LabView. The graphic interface consists of modulators to vary the amplitude and frequency of the hip and knee trajectories during the walking. The maximum amplitude and frequency are limited to avoid patient injury. Additionally, the prototype movement is restricted by mechanical limits.

The test consists of the following steps: First, the subject is attached to the exoskeleton using a crane. Second, the system is turned on and the therapist sets the values of the walking trajectory in the graphical user interface. Then, the therapist begins the system by clicking on the start button in the graphical interface. The system executes the walking movement until the operator clicks on the end button, and the system finishes the cycle and goes to the initial position. A constant disturbance is added to the prototype to prove the robustness of the controller.

**Figure 7:**
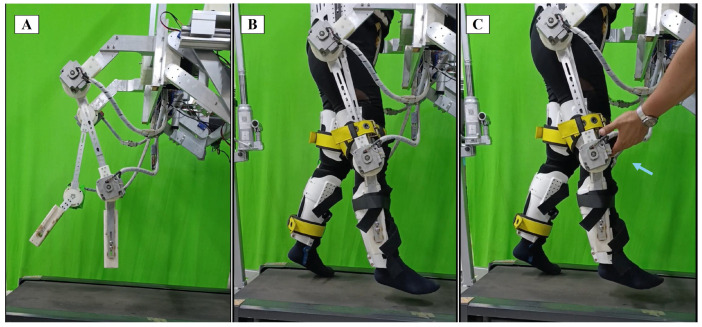
Experimental tests: (**A**) only exoskeleton, (**B**) exoskeleton + user, and (**C**) exoskeleton + user + external disturbance (blue arrow).

Three experimental tests were performed, so in experimental test A, the exoskeleton was used to follow the desired trajectory for gait rehabilitation (Figure 7A). In test B, the exoskeleton with a healthy user was evaluated to validate the robustness of the controller, considering the user as an unknown uncertainty (Figure 7B). In test C, external disturbances were added to the system and were rejected by the controller, so the robot followed the desired trajectory (Figure 7C).

### 4.3. Experimental Results

The experimental tests consist of specifying the setup of the walking cycle on the user interface following the steps in Section 4.2. The frequency and the amplitude of the trigonometric functions are established with a frequency of 0.15 Hz and an amplitude of 0.25. Moreover, the experimental values of the neural network are assigned as specified in the simulation section.

**Figure 8:**
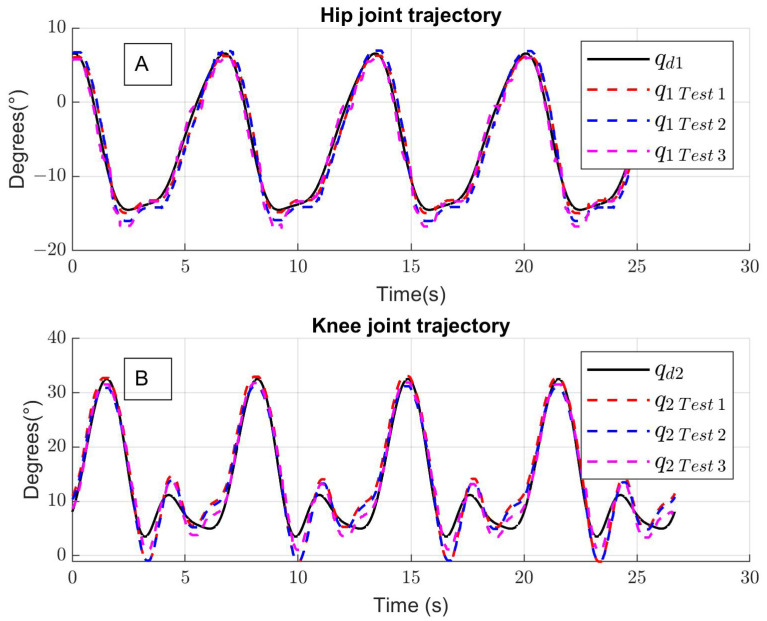
Experimental tracking trajectories (four walking cycles) for (**A**) hip joint and (**B**) knee joint.

Figure 8 shows the tracking trajectories of four walking cycles executed by the lower-limb exoskeleton using the experimental setup. The solid black line is the desired trajectory $q_{d1}$, the dotted red line shows the trajectory tracking of the hip robot joint $q_{1}$ for test 1 (only exoskeleton), the dotted blue line shows the trajectory tracking of the hip for test 2 (exoskeleton + user), and the dotted magenta line shows the trajectory tracking for test 3 (exoskeleton + user + disturbances).

**Figure 9:**
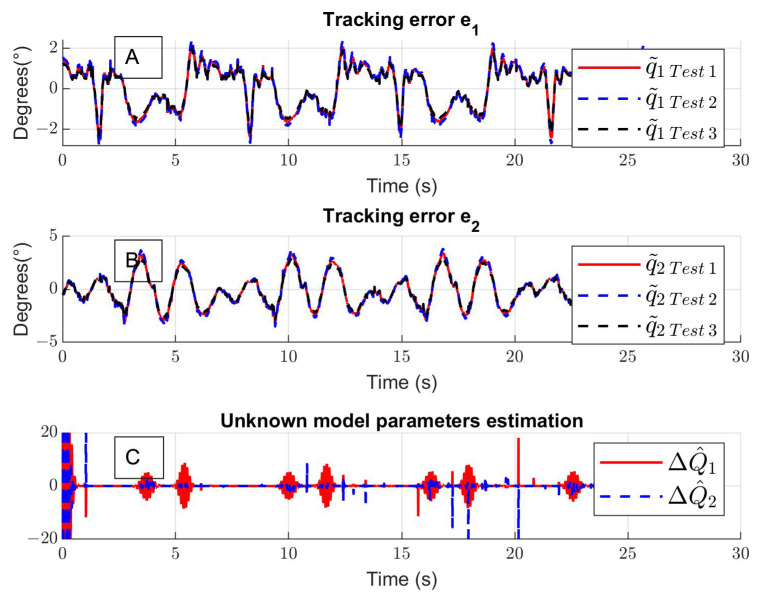
(**A**,**B**) Experimental tracking error for hip and knee joint and (**C**) unknown parameter estimation.

**Figure 10:**
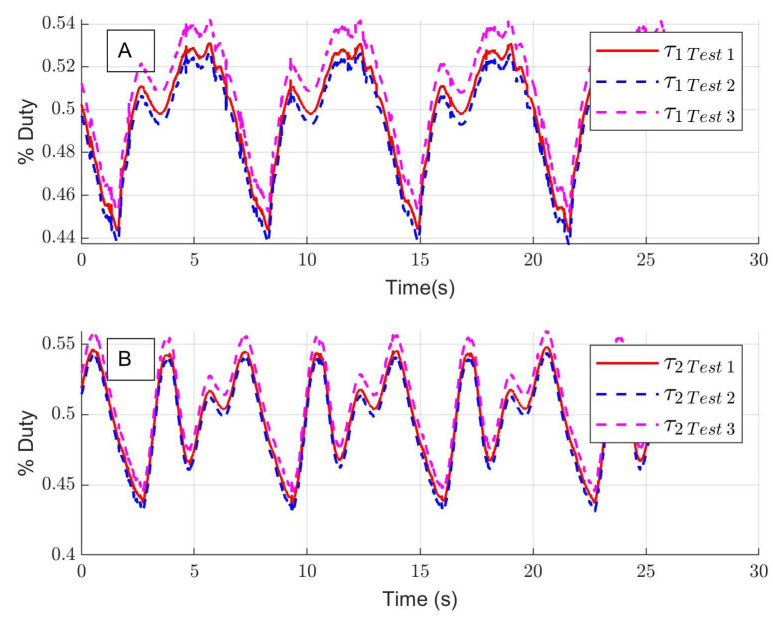
(**A**) Experimental control input for hip $\tau_{1}$; (**B**) experimental control input for knee $\tau_{2}$.

Figure 9A and Figure 9B present the tracking error for the hip and the knee, and the means of absolute errors are ${\tilde{q}}_{1}=1.27$° and ${\tilde{q}}_{2}=2.02$° for the hip and the knee, respectively. The estimated unknown function is shown in Figure 9C. The experimental control inputs for the hip $\tau_{1}$ and knee $\tau_{2}$ are shown in Figure 10A,B; the units are the percentage of duty cycle of the pulse width modulation.

The links for the videos of the experimental tests are listed as follows:

- In test A, the exoskeleton is used to follow the desired trajectory for gait rehabilitation (https://youtu.be/g3pz0G4x_aw accessed on 20 September 2024).
- In test B, the exoskeleton with a healthy user is tested to validate the robustness of the controller considering the user as an unknown uncertainty (https://youtu.be/r4ciHMjtOJo accessed on 20 September 2024.
- In test C, external disturbances are added to the system and are rejected by the controller, so the robot follows the desired trajectory (https://youtu.be/kjzzASk1yII accessed on 20 September 2024).

## 5. Conclusions

A control based on Artificial Neural Networks was applied to a system consisting of a robotic exoskeleton for gait rehabilitation. A leg exoskeleton with elastic joints was used to give comfort to the patient and to sense human intention of motion, as well as a linear feedback with an ANN estimator, which compensated the nonlinear dynamics and disturbances. This compensation provides robustness performance. The linear control guarantied an over-damping response for the tracking trajectory, which is important to smoothly follow the trajectories and avoid hurting the patient, during the therapy when external disturbances were present. The numerical simulation and the experimental results showed good performance of the tracking trajectory and validated the proposed control strategy. The robot joint trajectories converged to the desired trajectories, and tracking errors converged to a bounded region $\|e\|\;\leq \;2.82^{\circ }$ given by Equation (40), even when perturbations were included in the experimental setup.

The practical significance of the results is the relevance of the designed low-cost exoskeleton, which is capable of performing walking trajectories for people who have lower-limb disabilities. The test shows that the system is able to reject external disturbances and track the walking trajectories at a time response of less than 2 s. These characteristics guarantee security during rehabilitation even if there exist vibrations.

The further planned work is to sense the human intention of motion to activate the walking cycle and to perform active rehabilitation. Active rehabilitation means that the patient executes part of the effort to move the exoskeleton, and then the exoskeleton provides assistance as the patient needs it. Finally, we planned the validation of the system by a rehabilitation institute and the acceptance of a protocol to perform tests on injured patients.

## Data Availability

Data are contained within the article.
